# Illumination matters Part II: advanced comparative analysis of flexible ureteroscopes in a kidney model by PEARLS

**DOI:** 10.1007/s00345-024-04987-2

**Published:** 2024-05-06

**Authors:** Jia-Lun Kwok, Frédéric Panthier, Vincent De Coninck, Eugenio Ventimiglia, Yazeed Barghouthy, Alexandre Danilovic, Niamh Smyth, Jan Brachlow, Florian Alexander Schmid, Cédric Poyet, Daniel Eberli, Olivier Traxer, Etienne Xavier Keller

**Affiliations:** 1 Department of Urology, University Hospital Zurich, University of Zurich, Frauenklinikstrasse 10, 8091 Zurich, Switzerland; 2 Department of Urology, Tan Tock Seng Hospital, Singapore, Singapore; 3 Progressive Endourological Association for Research and Leading Solutions (PEARLS), Paris, France; 4 GRC N°20, Groupe de Recherche Clinique Sur La Lithiase Urinaire, Hôpital Tenon, Sorbonne Université, 75020 Paris, France; 5 Young Academic Urologists (YAU), Endourology & Urolithiasis Working Group, Arnhem, The Netherlands; 6 Department of Urology, AZ Klina, Brasschaat, Belgium; 7 Division of Experimental Oncology/Unit of Urology, Urological Research Institute, IRCCS Ospedale San Raffaele, Milan, Italy; 8 Department of Urology, Centre Hospitalier de Valenciennes, Valenciennes, France; 9 Department of Urology, Universidade de São Paulo Hospital das Clínicas-HCUSP, São Paulo, Brazil; 10 Department of Urology, Hospital Alemão Oswaldo Cruz, São Paulo, Brazil; 11 University Hospital Monklands, Monkscourt Avenue, Airdrie, ML60JS UK; 12 Zentrum Für Urologie Winterthur, Winterthur, Switzerland

**Keywords:** Flexible ureteroscopy, Light distribution, Illumination properties, Illuminance, Saline, Enclosed cavity

## Abstract

**Purpose:**

The aim of the study was to evaluate illumination properties in an in-vitro kidney calyx model in saline.

**Design and methods:**

We evaluated a series of contemporary flexible ureteroscopes including the Storz Flex-Xc and Flex-X2s, Olympus V3 and P7, Pusen 7.5F and 9.2F, as well as OTU WiScope using a 3D-printed closed pink kidney calyx model, submerged in saline. A spectrometer was used for illuminance and color temperature measurements at different openings located at center (direct light), 45° (direct and indirect light) and 90°(indirect light) to the axis of the scope.

**Results:**

Maximum illuminance was at the center opening for all scopes (range: 284 to 12,058 lx at 50% brightness and 454 to 11,871 lx at 100% brightness settings). The scope with the highest center illuminance (Flex-Xc) was 26 times superior to the scope with the lowest illuminance (Pusen 7.5Fr) at 100% brightness setting. For each scope, there was a peripheral illuminance drop ranging from − 43 to − 92% at 50% brightness and − 43% to − 88% at 100% brightness settings, respectively (all *p* < 0.01). Highest drop was for the P7 and the Pusen 9.2F. All scopes had illuminance skew, except the V3. All scopes had a warm color temperature.

**Conclusion:**

Illumination properties vary between ureteroscopes in an enclosed cavity in saline, and differs at center vs 45° and 90° positions within scopes. Peripheral illuminance drop can be as high as − 92%, which is undesirable. This may affect the choice of ureteroscope and light brightness settings used in surgery by urologists.

**Supplementary Information:**

The online version contains supplementary material available at 10.1007/s00345-024-04987-2.

## Introduction

The global prevalence of stone disease is increasing [1–3]. In tandem, there has been a huge increase in ureteroscopy procedures worldwide over the last two decades, which is currently the most frequent intervention for renal stones [2].

Clarity of vision during ureteroscopy is important and ensures accurate identification and precise targeting of pathology [4–8]. Visibility typically becomes of utmost relevance for tumor detection, safe stone lasering or future implementation of artificial intelligence [9, 10].

Many factors play a role in vision and clarity of images for ureteroscopy. These include illuminance and light distribution from the ureteroscope tip to the target object, and environmental factors such as the effects of direct and indirect light in an enclosed kidney cavity.

To the best of our knowledge, distribution of illuminance from light sources of ureteroscopes in an enclosed cavity in saline has not been evaluated so far. Of the prior studies that have evaluated light source of ureteroscopes, most were done in open systems [6, 7, 11–15]. Only one study in literature incorporated an enclosed system, but a diffuser plate was applied to the incoming light [16]. Another study looked at the effects of an integrating sphere on light transmission and measured only the integrated light output from one single opening [17]. No prior study looked at light measurements at different points in an enclosed sphere. More importantly, all these prior studies were performed in air, while illumination properties have been shown to be different with air and saline in a recent study, saline being the medium used in clinical routine during ureteroscopy [15].

The aim of the study was to evaluate illuminance and color temperature from ureteroscopy light sources within a closed in-vitro kidney calyx model in saline, including distribution of direct and indirect light.

## Material and methods

A series of ureteroscopes available at our institution were evaluated, including the Flex-Xc and Flex-X2s (Karl Storz SE & Co. KG, Tuttlingen Germany), the URF-P7 and URF-V3 (Olympus, Center Valley, PA, USA), and single use scopes Uscope 7.5F PU3033A, Uscope 9.2F PU3022A (Zhuhai Pusen Medical Technology Co. Ltd. Guangdong, China) and WiScope (OTU Medical Inc., CA, USA). The single use scopes (Pusen 7.5F, Pusen 9.2F, OTU WiScope) were brand new from sealed sterile packages, to reflect real world working conditions. Reusable scopes (Storz and Olympus scopes) had all undergone rinsing, disinfection, and decontaminated after clinical use, with no record of the number of prior case usage.

Together with the Storz Flex-X2s, the Power LED 175 light source (unit used < 100 h) was used with the corresponding 230 cm and 3.5 mm fiberoptic cable, as well as the IMAGE1 S HX-P HDTV 1-Chip pendular camera (Karl Storz SE & Co. KG, Tuttlingen, Germany). For the Olympus URF-P7 and URF-V3, the VISERA elite CLV-S190 light source (Xenon short-arc lamp used < 100 h) with the corresponding WA03310A 300 cm and 4.3 mm fiberoptic light cable, as well as the CH S190 08 LB camera head (Olympus, Center Valley, PA, USA) were used. The fiberoptic cables were brand new.

A color spectrometer housing the Vishay VEML 6040 color sensor (RGBW200, ELV Elektronik AG, Leer, Germany) was used for illuminance and color temperature measurements in saline, as previously described [15].

**Fig. 1:**
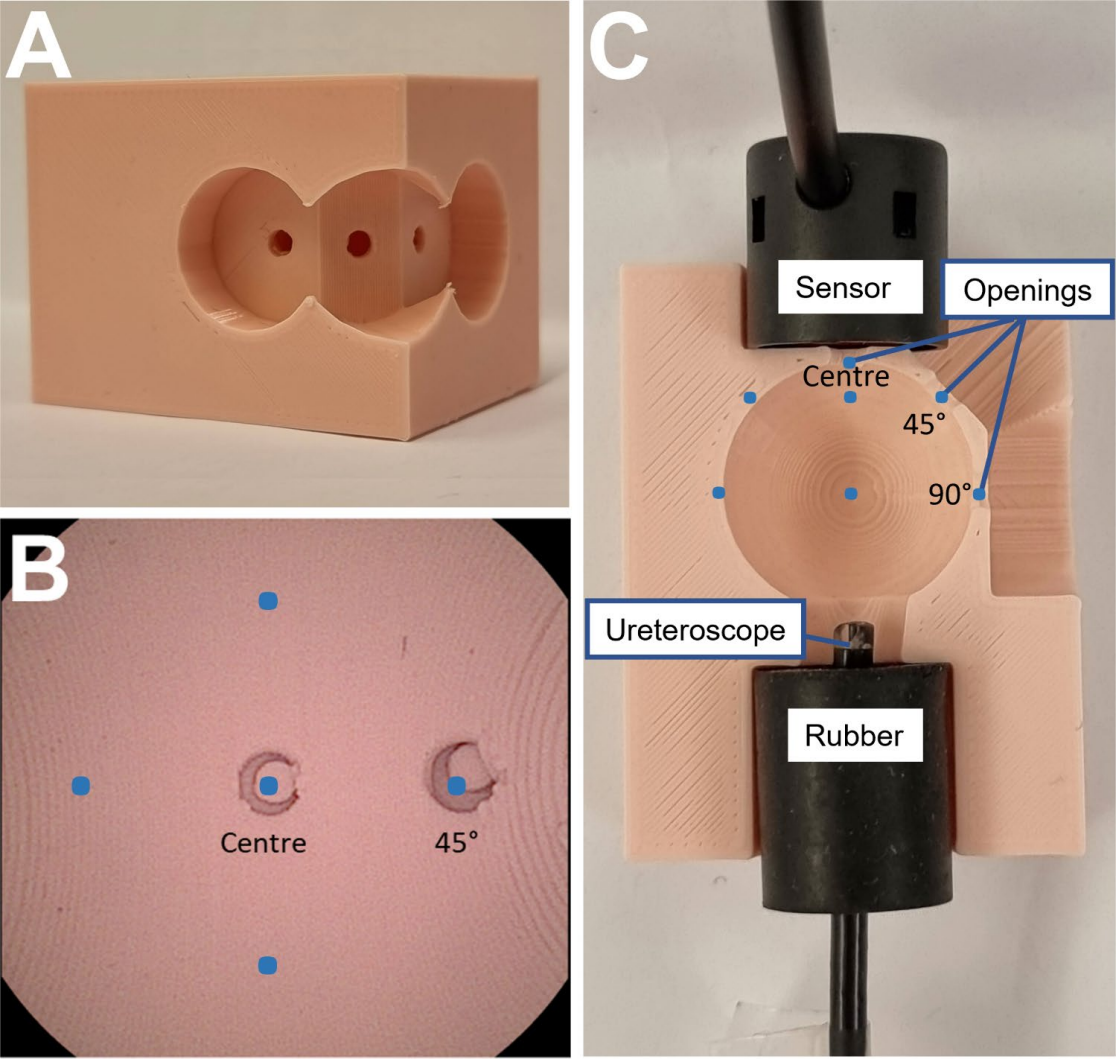
Experimental set-up. **A** Photograph of the 3D-printed enclosed pink model used for measurements with light measurement openings at 0°, 45° and 90 °C from the scope’s axis. The photograph is an outside view of the model. For measurements, obliterating 3D-printed pink counter-pieces were used to leave only one measurement opening accessible to the spectrometer’s sensor (not shown in this photograph to the sake of better representation of the openings from an outside view). **B** Endoscopic view of the 3D-printed enclosed pink model (still image from a Storz Flex-Xc inserted in the pink model, which was completely immerged in saline). The visible openings for the spectrometer sensor are obliterated with 3D-printed pink counter-pieces in this image, with the open 90° position out of the field of vision. Blue dots indicate the actual and virtual light measurement positions. The actual model was turned 90° relative to the axis of the scope to cover all virtual positions for each 45° and 90° opening positions. **C** Cross section of the 3D-printed pink model cut in half to display the position of the ureteroscope in relation to the spherical cavity and its opening positions for the spectrometer sensor. The black rubber cylinder was used to ensure solid tightening of the ureteroscope within the model during measurements. The tip of a OTU WiScope is visible and looking out 4 mm from the black rubber to ensure a standardized distance of 20 mm between tip of the scope and the concave surface of the calyx model. Blue dots indicate the actual and virtual light measurement positions. To cover all positions, the calyx model was turned in the axis of the scopes in successive 90° steps

A 3D-printed pink kidney calyx model was used to hold the ureteroscopes at a fixed distance of 20 mm from the concave surface of the calyx model in a dark room (Fig. 1). This consisted of a closed spherical cavity to replicate the human kidney calyx and included three openings located at the center, 45°and 90° to the axis of the scope. The model was rotated in successive 90° steps relative to the axis of the scope to obtain measurements at nine points across the sphere. Measurement openings were chosen to measure the direct light (center), combined direct and indirect light (45°) and indirect light only (90°). Peripheral illuminance and peripheral color temperature were defined as measurements from opening positions at 45° and 90°, as opposed to measurements from the center opening position. Pink material was chosen for the kidney calyx model to replicate human urothelial mucosa. The ureteroscope was kept in a non-deflected position, with the center of the scope view aligned to the center sensor opening. To reflect in vivo settings, the size of the target field and distance from the light sensor was chosen based on dimensions of models constructed on data from endocasts [18] used for in-vitro studies evaluating laser lithotripsy [19, 20]. All measurements were performed in saline to replicate the usual conditions found in ureteroscopy clinical routine.

For each measurement, the color spectrometer sensor was placed at one of the nine opening positions. Five repeated measurements were taken at each position. For each measurement, the ureteroscope was withdrawn and reinserted in the kidney calyx model. Illuminance (lux) and color temperature (Kelvin) were measured at all nine opening positions.

When the scope set-up allowed light source brightness adjustment, measurements were done at light brightness settings of 50% and 100%. This was found for the Storz Flex-Xc to be adjusted only via the menu buttons on the scope handle. For the Storz Flex-X2s, in addition to brightness settings adjustable on the light source unit, buttons on the camera head also allowed for separate brightness adjustment. Based on our findings with the Storz Flex-Xc, brightness settings for the Storz Flex-X2s in the camera head was kept at 50% and brightness settings only adjusted on the light source unit. The brightness setting for all other scopes was adjusted on the light source unit only. As brightness can be set to automatic or manual mode on the Olympus light stack as well as on the Storz camera head, we performed all measurements using the manual mode to ensure consistency throughout all experiments.

### Statistical analysis

Illuminance and color temperature for a given opening position were expressed as mean values of five repeated measurements. Overall illuminance and overall color temperature were defined as mean from all measurement opening positions for a given scope. Illuminance skew was defined as the direction of the highest peripheral illuminance (mean of 45° and 90° position) in a clock face fashion for a given scope. Any other direction with no statistically significant difference compared to that highest peripheral illuminance direction also accounted for illuminance skew. To quantify the extent of skewness for a given scope, the lowest peripheral illuminance direction was considered and reported as percentage relative to the highest peripheral illuminance direction. This reflects the relative intra-scope dominant direction(s) of peripheral illuminance and, therefore, skewness of the scopes, with comparison to the areas of possible relative darkness to quantify the extent of skewness.

Comparisons of continuous values over two groups were evaluated by Student’s t tests. Comparisons of continuous values over multiple groups were evaluated by one-way ANOVA with Tukey post hoc comparisons. All statistical tests were performed with GraphPad Prism 9.5.1 (GraphPad Software, La Jolla CA, USA). Radar charts of the light illuminance skewness were made with Microsoft Excel for Microsoft 365 MSO Version 2302 Build 16.0.16130.20332 (Microsoft Corporation, Redmond WA, USA).

## Results

### Overall illuminance

Overall inter-scope illuminance significantly differed between flexible ureteroscopes for both 50% and 100% brightness settings (both ANOVA *p* < 0.001) (Table 1).

**Table 1:** Comparison of overall illuminance of flexible ureteroscopes in a pink kidney calyx model

Scope	Illuminance				
	Mean overall illuminance* at 50% brightness setting (lux) (95% CI)	Mean overall illuminance* at 100% brightness setting (lux) (95% CI)	Mean increase (lux) (95% CI)	Percentage increase (%)	*p* value**
Reusable					
Storz Flex-Xc	5858 (5145 to 6570)	5890 (5194 to 6586)	33 (− 949 to 1015)	1	*p* = 0.95
Storz Flex-X2s	1874 (1474 to 2275)	2596 (2069 to 3123)	722 (69 to 1375)	39	p = 0.03
Olympus V3	345 (232 to 458)	1422 (996 to 1848)	1077 (643 to 1512)	312	p < 0.001
Olympus P7	1317 (748 to 1887)	2512 (1676 to 3348)	1195 (197 to 2192)	91	p = 0.02
Single-use					
Pusen 7.5F	169 (154 to 183)	272 (248 to 296)	103 (76 to 131)	61	p < 0.001
Pusen 9.2F	1341 (836 to 1845)	2093 (1347 to 2839)	753 (− 136 to 1640)	56	p = 0.10
OTU WiScope	285 (259 to 310)	563 (502 to 624)	278 (214 to 343)	98	p < 0.001

^*^ Considering all measurements over all target board openings

^**^ Student’s *t* test comparing illuminance at 50% vs. 100%

The Flex-Xc was the scope with the highest overall illuminance at 100% brightness setting (5890 lx), followed by Flex-X2s (2596 lx), P7 (2512 lx), Pusen 9.2F (2093 lx), V3 (1422 lx), WiScope (563 lx) and finally the Pusen 7.5F with the lowest overall illuminance (272 lx). This same ranking was found at 50% brightness setting (Table 1).

Intra-scope comparison between the 50% and the 100% brightness setting revealed significant differences in overall illuminance for all scopes (all *p* < 0.05), except for the Flex-Xc (*p* = 0.95) and Pusen 9.2F (*p* = 0.10). The highest relative intra-scope illuminance increase between 50 and 100% brightness setting was for the V3 (+ 312%), and the lowest illuminance increase for the Flex-Xc (+ 1%).

**Fig. 2:**
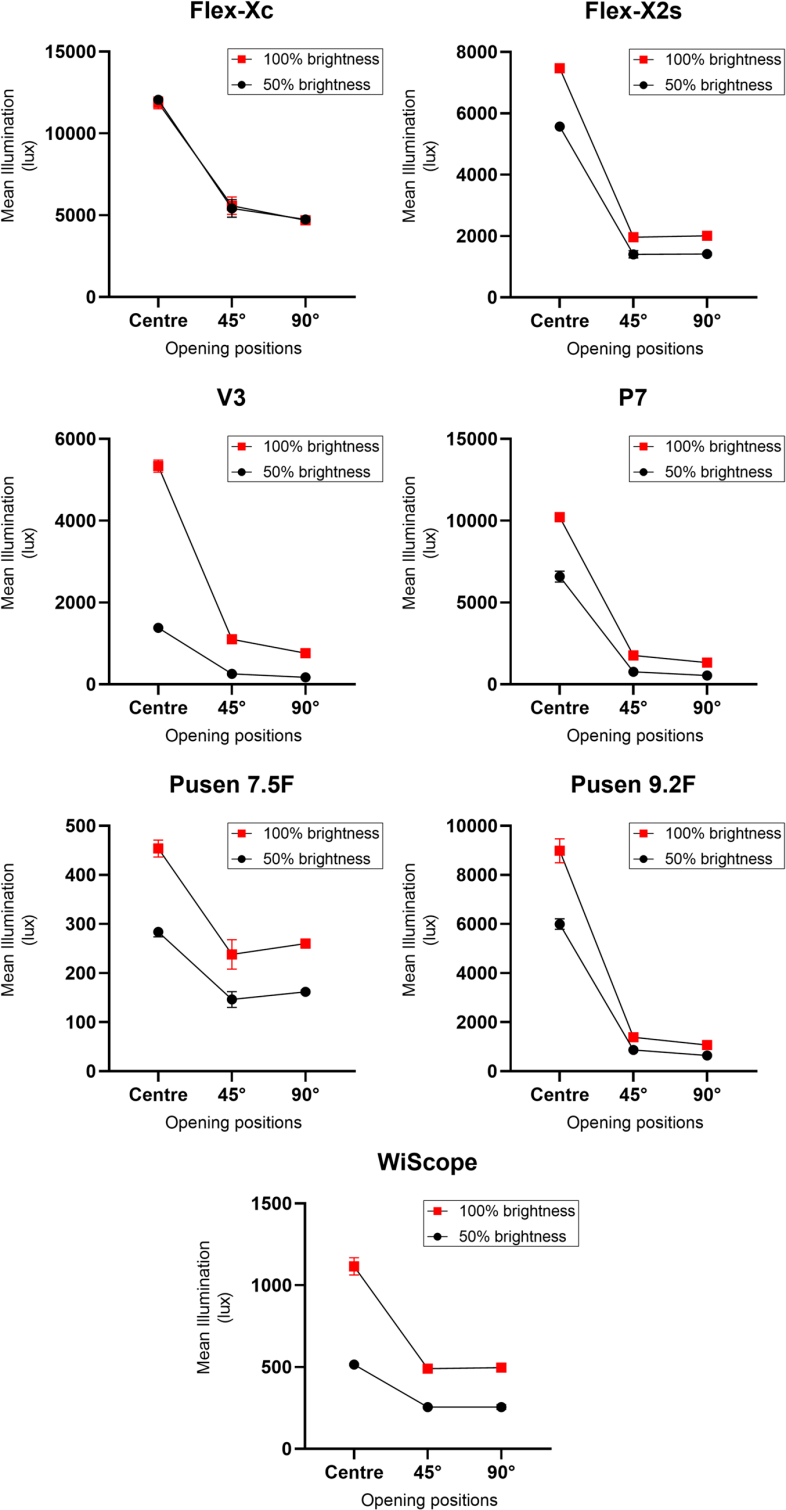
Comparison of center versus peripheral illuminance of flexible ureteroscopes. Data are mean illuminance at center, 45°and 90° opening positions with 95% CI measured in an enclosed pink kidney calyx model. The Y-axes scales are different for each graph

When considering each opening position separately, maximum illuminance was found at the center opening for all scopes (ranging 516–12,058 lx at 50% brightness and 454–11,871 lx at 100% brightness settings), with a consistent drop in illuminance from the center to 45° and 90° opening positions (peripheral illuminance drop) (Table 2, Fig. 2).

**Table 2:** Direct and indirect light effects on illuminance in a pink kidney calyx model

Scope	Mean illuminance (lux)																			
	50% brightness setting										100% brightness setting									
	Centre (95% CI)	Centre relative factor to lowest illuminance scope	45° opening positions (95% CI)	90° opening positions (95% CI)	Mean change center vs 45° (95% CI)	% change	*p* value*	Mean change center vs 90° (95% CI)	% change	*p* value**	Centre (95% CI)	Centre relative factor to lowest illuminance scope	45° opening positions (95% CI)	90° opening positions (95% CI)	Mean change center vs 45° (95% CI)	% change	*p* value*	Mean change center vs 90° (95% CI)	% change	*p* value**
Storz Flex-Xc	12,058 (11,807 to 4798)	42 times	5420 (4883 to 5957)	4745 (4633 to 4868)	− 6638 (− 7721 to − 5556)	− 55%	*p* < 0.01	− 7313 (− 7575 to − 7051)	− 61%	*p* < 0.0.1	11,871 (11,508 to 12,235)	26 times	5586 (5053 to 6119)	4699 (4551 to 4848)	− 6286 (− 7364 to − 5207)	− 53%	*p* < 0.01	− 7172 (− 7497 to − 6847)	− 60%	*p* < 0.01
Storz Flex-X2s	5570 (5414 to 5726)	20 times	1406 (1287 to 1526)	1418 (1375 to 1461)	− 4164 (− 4409 to − 3918)	− 75%	*p* < 0.01	− 4152 (− 4254 to − 4050)	− 75%	*p* < 0.01	7470 (7413 to 7527)	16 times	1966 (1832 to 2099)	2008 (1948 to 2068)	− 5504 (− 5773 to − 5235)	− 74%	*p* < 0.01	− 5462 (− 5585 to − 5339)	− 74%	*p* < 0.01
Olympus V3	1384 (1301 to 1466)	5 times	257 (232 to 281)	174 (161 to 187)	− 1127 (− 1184 to − 1070)	− 81%	*p* < 0.01	− 1210 (− 1249 to − 1171)	− 87%	*p* < 0.01	5336 (5189 to 5482)	12 times	1101 (1003 to 1200)	765 (708 to 821)	− 4234 (− 4438 to − 4030)	− 79%	*p* < 0.01	− 4571 (− 4695 to − 4447)	− 86%	*p* < 0.01
Olympus P7	6590 (6259 to 6922)	23 times	774 (688 to 860)	542 (496 to 587)	− 5816 (− 6024 to − 5609)	− 88%	*p* < 0.01	− 6049 (− 6196 to − 5902)	− 92%	*p* < 0.01	10,215 (10,104 to 10,326)	23 times	1770 (1553 to 1988)	1328 (1225 to 1431)	− 8444 (− 8883 to − 8006)	− 83%	*p* < 0.01	− 8887 (− 9098 to − 8676)	− 87%	*p* < 0.01
Pusen 7.5F	284 (274 to 294)	-	146 (130 to 162)	162 (159 to 165)	− 138 (− 170 to − 105)	− 49%	*p* < 0.01	− 122 (− 129 to − 114)	− 43%	*p* < 0.01	454 (437 to 471)	–	238 (208 to 268)	260 (255 to 266)	− 216 (− 276 to − 155)	− 48%	*p* < 0.01	− 194 (− 206 to − 181)	− 43%	*p* < 0.01
Pusen 9.2F	5999 (5784 to 6214)	21 times	869 (740 to 997)	649 (634 to 664)	− 5130 (− 5399 to − 4861)	− 86%	*p* < 0.01	− 5350 (− 5430 to − 5269)	− 89%	*p* < 0.001	8986 (8498 to 9473)	20 times	1391 (1226 to 1556)	1073 (1048 to 1098)	− 7595 (− 7967 to − 7223)	− 85%	*p* < 0.01	− 7913 (− 8089 to − 7736)	− 88%	*p* < 0.01
OTU WiScope	516 (503 to 529)	2 times	255 (249 to 262)	256 (242 to 270)	− 261 (− 274 to − 248)	− 51%	*p* < 0.01	− 260 (− 289 to − 231)	− 50%	*p* < 0.01	1115 (1063 to 1168)	2 times	491 (477 to 504)	497 (473 to 521)	− 625 (− 657 to − 592)	− 56%	*p* < 0.01	− 618 (− 670 to − 566)	− 55%	*p* < 0.01

^*^Student’s *t* test comparing center vs 45°

^**^Student’s *t* test comparing center vs 90°

### Illuminance at center opening position

Illuminance at the center opening position significantly differed between flexible ureteroscopes at both 50% and 100% brightness settings (both ANOVA *p* < 0.001). Tukey’s post hoc analysis revealed that mean differences between each scope were significant compared to any other reference scope at 50% and 100% brightness settings, except for the Pusen 7.5F and OTU WiScope at 50% brightness settings (Table 2).

When considering only the center opening instead of overall illuminance, the order of illuminance changed with the Flex-X2s falling two marks in the ranking. The Flex-Xc was still the scope with highest illuminance at 100% (center illuminance 11,871 lx), followed by P7 (10,215 lx), Pusen 9.2F (8986 lx), Flex-X2s (7470 lx), V3 (5336 lx), WiScope (1115 lx) and finally the Pusen 7.5F with the lowest illuminance readings (454 lx). The same ranking was found for 50% brightness settings. The scope with highest illuminance (Flex-Xc) had 42 times and 26 times higher illuminance compared to the scope with lowest illuminance (Pusen 7.5Fr), at 50% and 100% brightness settings, respectively.

### Peripheral illuminance

There was a significant peripheral illuminance drop for all scopes at both 50% and 100% brightness settings (all *p* < 0.01) (Table 2) (Fig. 2). When comparing illuminance at center vs 45° opening positions, the difference ranged from − 49 to − 88% at 50% brightness setting and − 48% to − 85% at 100% brightness setting (all *p* < 0.01). When comparing illuminance at center vs 90° opening positions, the difference ranged from − 43 to − 92% at 50% brightness setting and − 43% to − 88% at 100% brightness setting (all *p* < 0.01).

The scopes with the highest peripheral illuminance drop were the P7 (− 88% for center vs 45° and − 92% for center vs 90° at 50% brightness setting; − 83% and − 87% at 100% brightness setting) and the Pusen 9.2F (− 86% and − 89% at 50% brightness setting; − 85% and − 88% at 100% brightness setting). The scopes with the least peripheral illuminance drop were the Pusen 7.5F (− 49% and − 43% at 50% brightness setting; − 48% and − 43% at 100% brightness setting) and the WiScope (− 51% and − 50% at 50% brightness setting; − 56% and − 55% at 100% brightness setting).

### Illuminance skew

**Fig. 3:**
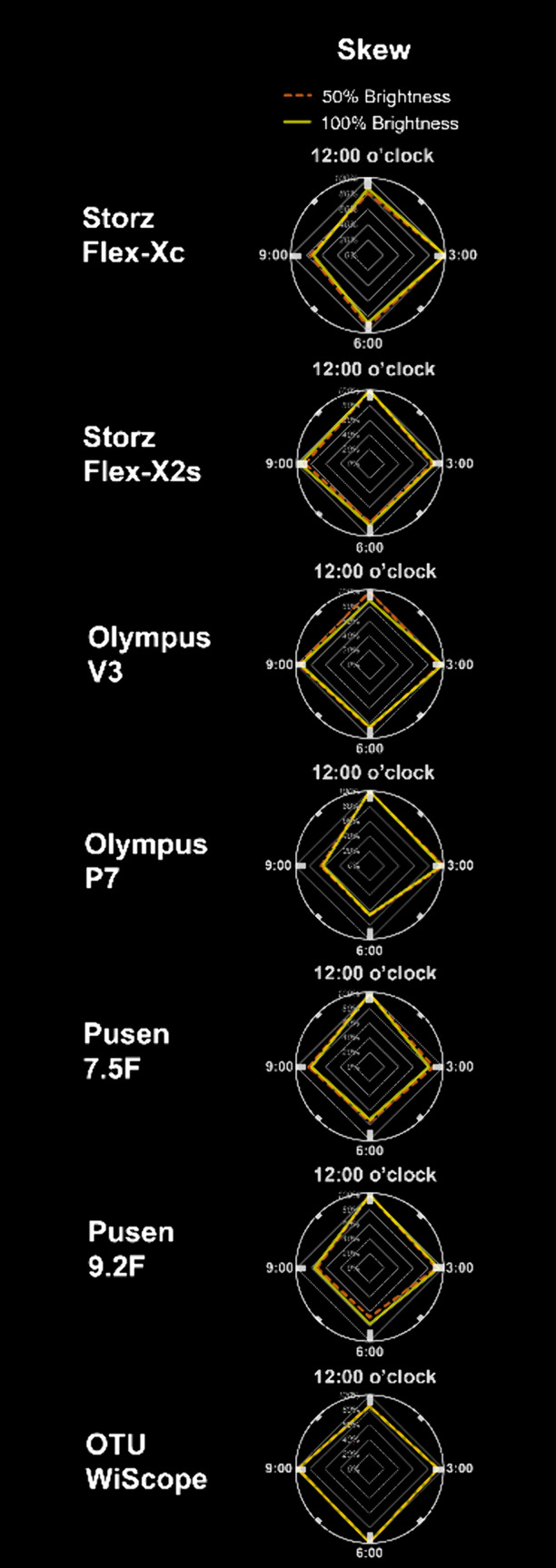
Illuminance skew of flexible ureteroscopes in a pink kidney calyx model under saline. Skew of ureteroscopes are represented by radar charts, comparing mean peripheral illuminance (45° and 90° opening positions) at each 9, 12, 3 and 6 o’clock directions, expressed as percentage of illuminance from the maximum direction. Exact values are found in Supplementary Table S1

There was a significant illuminance skew for all scopes (ANOVA *p* < 0.001), except for the V3 (Fig. 3) (Supplementary Table S1). The scope with the highest illuminance skewness was the P7 with dark areas of illuminance as low as 62% relative to the highest peripheral illuminance direction, followed by the Pusen 9.2 F (67%), Flex-Xc (71%), Pusen 7.5F (72%), Flex-X2s (80%), WiScope (85%).

### Color temperature

Inter-scope comparisons revealed significantly different overall color temperatures at both 50% and 100% brightness settings (ANOVA *p* < 0.001) (Supplementary Fig. S1, Supplementary Table S2). The two scopes with the warmest (lowest Kelvin values) overall color temperatures were the V3 and P7 (range 2922 to 3160 Kelvin). The two scopes with the coolest (highest Kelvin values) overall color temperatures were the Pusen 7.5F and PUSEN 9.2F (range 4045 to 4592 Kelvin).

Intra-scope comparison between the 50% and the 100% brightness setting revealed no significant difference in overall color temperature for any scope (all scopes *p* > 0.05).

For each scope, the center opening always had cooler color temperatures compared to the periphery, with a peripheral drop to warmer (lower) Kelvin values. At 50% brightness setting, the difference of color temperature between the position “center” vs. “45°” ranged from − 24 to − 50%, and “center” vs. “90°” from − 27% to − 55%. At 100% brightness setting, “center” vs. “45°” ranged from − 23 to − 44%, and “center” vs. “90°” from − 31 to − 50% (all *p* < 0.01).

## Discussion

Overall illuminance varied widely between flexible ureteroscopes. More importantly, all ureteroscopes showed significant peripheral illuminance drops as well as significant illuminance skew.

Maximum illuminance for all ureteroscopes in this study was at the center of the 3D kidney calyx model. Prior evaluation in a non-enclosed system had shown that the maximum illuminance for the same set of ureteroscopes was actually mostly off center [15]. This underlines the importance of the present evaluation in an enclosed spherical pink kidney calyx model, where the effects of direct and indirect light arguably resulted in maximum illuminance readings at the center opening for all scopes, akin to an integrating sphere. This finding also seems to be independent from the various number of light sources and relative positions thereof on the ureteroscope tip that had been described before [15]. The center opening illuminance is the most clinically relevant in endourology, as most surgeons would by default aim to keep the target of interest in the center of the endoscopic field of view. This finding has potential implications on ureteroscope tip design for future developments in the field of endoscopy.

It is striking that all scopes suffered from a significant peripheral illuminance drop as high as − 92% between the center and 45° or 90° opening positions. This varied between ureteroscopes. In endourology, the target of interest may inadvertently be off center despite the best efforts of the surgeon, usually due to limitations of deflection combined with environmental situations in the kidney. In these situations, it is desirable to have a scope with as little peripheral drop as possible. Peripheral illuminance drop may become particularly problematic for scopes with a wide angle lens (“fisheye lens”), since such scopes would suffer from particularly poor peripheral illuminance compared to the center of the field of view [8].

For non-center illuminance, urologists need to be aware of the illuminance skew and potentially darker areas for each scope. This has possible implications on visibility in different parts of the visual field, especially in situations of poorer visibility due to bleeding, larger hydronephrotic systems, or working at further distances. While illuminance skew was present for most scopes, the enclosed calyceal system seems to have mitigated some of the more pronounced illuminance skew that was found in a prior analysis in a black non-enclosed model [15].

For two out of the seven scopes, no significant change in overall illuminance was found between 50 and 100% brightness settings: the Flex-Xc and the Pusen 9.2F. For the Flex-Xc, this absence of difference is explained by the fact that regulating brightness settings from 50 to 100% only impacts on the post-processing of the image, and not on the light source per se, as described before [15]. For the Pusen 9.2F, the high peripheral illuminance drop resulted in a larger range and distribution of illuminance readings from the nine opening positions of the model, resulting in larger 95% confidence intervals for the overall illuminance calculations and, therefore, not reaching the significance level when comparing 50% and 100% brightness settings.

The Kelvin scale for color temperature ranges from warm (lower value) to cool (higher value), and all the ureteroscopes were found within the warmer part of the Kelvin scale, in comparison to the readings in a prior study in a non-enclosed system [15]. This is arguably explained by the difference in light reflection and absorption between the pink 3D kidney calyx model of the present study and the black model that was used in our prior study. Whether warmer or cooler color temperature is more favorable in various situations could be further explored in future studies. The peripheral drop in color temperature when compared to the center opening measurements underlines the validity of the current model which seems to adequately differentiate direct light (center opening position) from combined direct and indirect light (45°) and indirect light only (90°).

Some potential limitations need to be addressed. First, the present study is an in-vitro attempt to assess ureteroscope properties that may impact on in vivo settings and interpretation of the results must be done carefully. Compared to the 3D-printed model in this study, the mucosa of human kidney collecting systems may vary in exact shade of pink, with additional impact of underlying structures such as blood vessels, and possibly different absorption and reflection properties. Second, environmental factors arguably may impact on clinical translation of the findings of this study. For example, blood [21], urine and stone dust may substantially impact on illuminance characteristics during surgery, and should be further evaluated in future studies. Third, there may be some variations to the actual size of the calyceal cavity during ureteroscopy. Fourth, the reusable scopes used in the present study were not brand new, with possible degradation of the optical systems (illumination and imaging [22]) with prior usage. However, this reflects real-world clinical conditions where reusable scopes are barely ever used brand new. Finally, it is not clear how illuminance properties may impact on the final video projection of the surgical site from ureteroscopes on operative monitors.

Future studies should evaluate how environmental factors including blood, urine and stone dust may affect the illumination properties of flexible ureteroscopes, including effects of partial light obstruction in an enclosed cavity, and further explore how our findings may impact on overall efficiency and safety aspects of the surgery, as well as on image-based technologies such as artificial intelligence in ureteroscopy [9, 10].

## Conclusions

Illumination properties vary widely between ureteroscopes within an enclosed cavity in saline, and for each scope illuminance differs as well at center vs 45° and 90° positions. Scopes can have a peripheral illuminance drop as high as − 92% compared to the center and the presence of illuminance skew, which are undesirable properties possibly impacting diagnostic purposes in ureteroscopy. Urologists should be aware of this as it may affect the choice of ureteroscopes, and corresponding choice of light brightness setting used in surgery.

## Supplementary Information

Below is the link to the electronic supplementary material.

Supplementary file1 (TIF 15854 KB). Color temperature of ureteroscopes in pink kidney calyx model. Color temperature measurements of ureteroscopes on a background representing the range of color temperatures. Lower Kelvin values are warmer colors (orange), and higher Kelvin values cooler colors (light blue).

Supplementary file2 (DOCX 17 KB)

Supplementary file3 (DOCX 18 KB)

## Data Availability

On request to corresponding author for raw data on the experimental setup.

## References

[1] Heers H, Stay D, Wiesmann T, Hofmann R (2022) Urolithiasis in Germany: trends from the National DRG database. Urol Int 106(6):589–595. 10.1159/000520372 34883491 10.1159/000520372 PMC9248299

[2] Geraghty RM, Jones P, Somani BK (2017) Worldwide trends of urinary stone disease treatment over the last two decades: a systematic review. J Endourol 31(6):547–556. 10.1089/end.2016.0895 28095709 10.1089/end.2016.0895

[3] Edvardsson VO, Indridason OS, Haraldsson G, Kjartansson O, Palsson R (2013) Temporal trends in the incidence of kidney stone disease. Kidney Int 83(1):146–152. 10.1038/ki.2012.320 22992468 10.1038/ki.2012.320

[4] Proietti S, Dragos L, Molina W, Doizi S, Giusti G, Traxer O (2016) Comparison of new single-use digital flexible ureteroscope versus nondisposable fiber optic and digital ureteroscope in a cadaveric model. J Endourol 30(6):655–659. 10.1089/end.2016.0051 27084572 10.1089/end.2016.0051 PMC4913498

[5] Lusch A, Abdelshehid C, Hidas G, Osann KE, Okhunov Z, McDougall E et al (2013) In vitro and in vivo comparison of optics and performance of a distal sensor ureteroscope versus a standard fiberoptic ureteroscope. J Endourol 27(7):896–902. 10.1089/end.2013.0003 23402369 10.1089/end.2013.0003 PMC3826566

[6] Hendriks N, Henderickx MMEL, Schout BMA, Baard J, van Etten-Jamaludin FS, Beerlage HP et al (2021) How to evaluate a flexible ureterorenoscope? Systematic mapping of existing evaluation methods. BJU Int 128(4):408–423. 10.1111/bju.15544 34242475 10.1111/bju.15544 PMC8519042

[7] Bader MJ, Gratzke C, Walther S, Schlenker B, Tilki D, Hocaoglu Y et al (2010) The polyScope: a modular design, semidisposable flexible ureterorenoscope system. J Endourol 24(7):1061–1066. 10.1089/end.2010.0077 20575699 10.1089/end.2010.0077

[8] Dragos LB, Somani BK, Keller EX, De Coninck VMJ, Herrero MRM, Kamphuis GM et al (2019) Characteristics of current digital single-use flexible ureteroscopes versus their reusable counterparts: an in-vitro comparative analysis. Transl Androl Urol 8:S359–S370. 10.21037/tau.2019.09.17 31656742 10.21037/tau.2019.09.17 PMC6790413

[9] Zeeshan Hameed BM, Aiswarya Dhavileswarapu VLS, Raza SZ, Karimi H, Khanuja HS, Shetty DK et al (2021) Artificial intelligence and its impact on urological diseases and management: a comprehensive review of the literature. J Clin Med. 10.3390/jcm10091864 10.3390/jcm10091864 PMC8123407 33925767

[10] Estrade V, Daudon M, Richard E, Bernhard JC, Bladou F, Robert G et al (2022) Towards automatic recognition of pure and mixed stones using intra-operative endoscopic digital images. BJU Int 129(2):234–242. 10.1111/bju.15515 34133814 10.1111/bju.15515 PMC9292712

[11] Patil A, Agrawal S, Batra R, Singh A, Ganpule A, Sabnis R et al (2023) Single-use flexible ureteroscopes: comparative in vitro analysis of four scopes. Asian J Urol 10(1):64–69. 10.1016/j.ajur.2022.02.001 36721687 10.1016/j.ajur.2022.02.001 PMC9875117

[12] Deininger S, Haberstock L, Kruck S, Neumann E, da Costa IA, Todenhöfer T et al (2018) Single-use versus reusable ureterorenoscopes for retrograde intrarenal surgery (RIRS): systematic comparative analysis of physical and optical properties in three different devices. World J Urol 36(12):2059–2063. 10.1007/s00345-018-2365-9 29869701 10.1007/s00345-018-2365-9

[13] Afane JS, Olweny EO, Bercowsky E, Sundaram CP, Dunn MD, Shalhav AL et al (2000) Flexible ureteroscopes: a single center evaluation of the durability and function of the new endoscopes smaller than 9Fr. J Urol 164(4):1164–1168. 10.1016/S0022-5347(05)67133-9 10992358

[14] Wilson CR, Kennedy JD, Irby PB, Fried NM (2018) Miniature ureteroscope distal tip designs for potential use in thulium fiber laser lithotripsy. J Biomed Opt. 10.1117/1.JBO.23.7.076003 29981222 10.1117/1.JBO.23.7.076003

[15] Kwok J-L, De Coninck V, Corrales M, Sierra A, Panthier F, Ventimiglia E, et al. Illumination matters Part I: comparative analysis of light sources and illumination in flexible ureteroscopy—fundamental findings from a PEARLS analysis. Undergoing peer review. Not yet published. 10.1007/s00345-024-05037-7 PMC11128383 38796790

[16] Paffen MLJE, Keizer JG, De Winter GV, Arends AJ, Hendrikx AJM (2008) A comparison of the physical properties of four new generation flexible ureteroscopes: (de)flection, flow properties, torsion stiffness, and optical characteristics. J Endourol 22(10):2227–2234. 10.1089/end.2008.0371 18831670 10.1089/end.2008.0371

[17] Abdelshehid C, Ahlering MT, Chou D, Park HK, Basillote J, Lee D et al (2005) Comparison of flexible ureteroscopes: deflection, irrigant flow and optical characteristics. J Urol 173(6):2017–2021. 10.1097/01.ju.0000158139.65771.0a 15879808 10.1097/01.ju.0000158139.65771.0a

[18] Marroig B, Favorito LA, Fortes MA, Sampaio FJB (2015) Lower pole anatomy and mid-renal-zone classification applied to flexible ureteroscopy: experimental study using human three-dimensional endocasts. Surg Radiol Anat 37(10):1243–1249. 10.1007/s00276-015-1503-y 26044783 10.1007/s00276-015-1503-y

[19] Aldoukhi AH, Roberts WW, Hall TL, Teichman JMH, Ghani KR (2018) Understanding the popcorn effect during holmium laser lithotripsy for dusting. Urology 122:52–57. 10.1016/j.urology.2018.08.031 30195011 10.1016/j.urology.2018.08.031

[20] Aldoukhi AH, Hall TL, Ghani KR, Roberts WW (2021) Strike rate: analysis of laser fiber to stone distance during different modes of laser lithotripsy. J Endourol 35(3):355–360. 10.1089/end.2020.0298 32631082 10.1089/end.2020.0298

[21] Talso M, Proietti S, Emiliani E, Gallioli A, Dragos L, Orosa A et al (2018) Comparison of flexible ureterorenoscope quality of vision: an in vitro study. J Endourol 32(6):523–528. 10.1089/end.2017.0838 29562765 10.1089/end.2017.0838

[22] Traxer O, Dubosq F, Jamali K, Gattegno B, Thibault P (2006) New-generation flexible ureterorenoscopes are more durable than previous ones. Urology 68(2):276–279. 10.1016/j.urology.2006.02.043 16904434 10.1016/j.urology.2006.02.043

