# Supplementary figures and images for: Illumination matters Part II: advanced comparative analysis of flexible ureteroscopes in a kidney model by PEARLS
Source: World J Urol. 2024 May 6;42(1):298. doi: 10.1007/s00345-024-04987-2 (PMC11074033; doi:10.1007/s00345-024-04987-2)

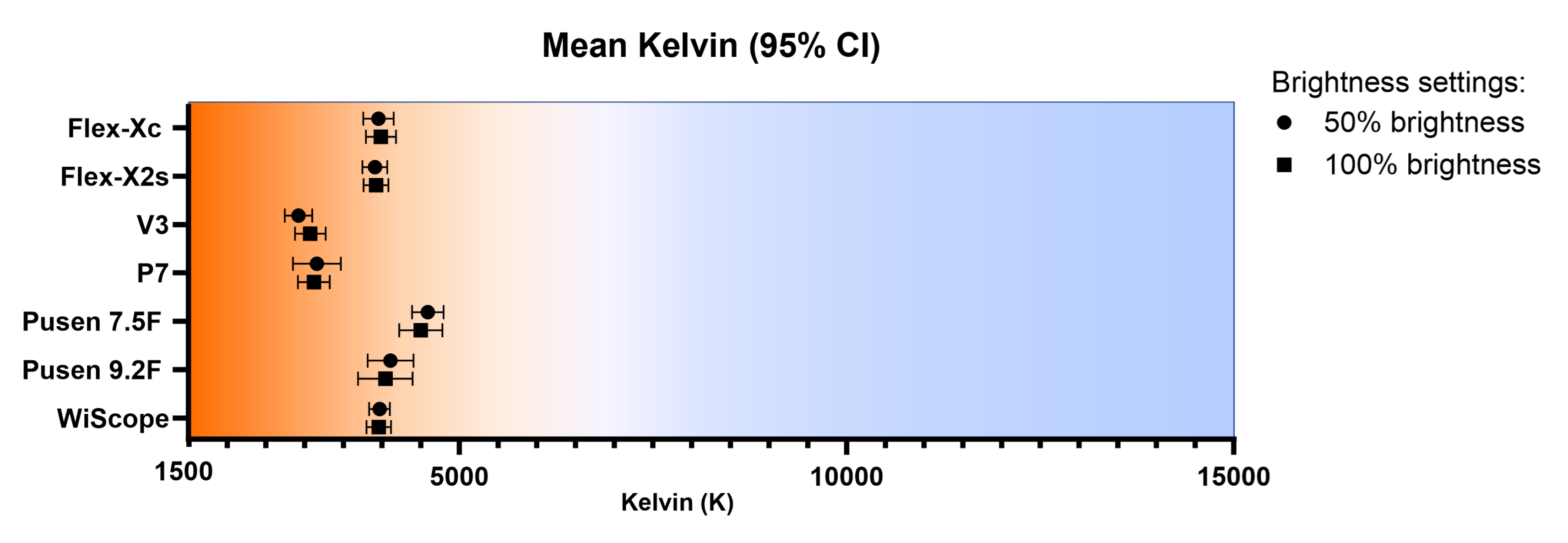

Supplement: Supplementary file 1 — Supplementary file1 (TIF 15854 KB). Color temperature of ureteroscopes in pink kidney calyx model. Color temperature measurements of ureteroscopes on a background representing the range of color temperatures. Lower Kelvin values are warmer colors (orange), and higher Kelvin values cooler colors (light blue). [file 345_2024_4987_MOESM1_ESM.tif]
