# Supplementary material for: Illumination matters Part II: advanced comparative analysis of flexible ureteroscopes in a kidney model by PEARLS
Source: World J Urol. 2024 May 6;42(1):298. doi: 10.1007/s00345-024-04987-2 (PMC11074033; doi:10.1007/s00345-024-04987-2)
Supplement: Supplementary file 2 — Supplementary file2 (DOCX 17 KB) [file 345_2024_4987_MOESM2_ESM.docx]

| **Supplementary Table 1. Illuminance skew of flexible ureteroscopes in a pink kidney calyx model** | | | | | | | | | | | | | | | | | | | | |
| --- | --- | --- | --- | --- | --- | --- | --- | --- | --- | --- | --- | --- | --- | --- | --- | --- | --- | --- | --- | --- |
| Scope | Mean peripheral illuminance (lux) | | | | | | | | | | | | | | | | | | | |
|  | 50% brightness setting | | | | | | | | | | 100% brightness setting | | | | | | | | | |
|  | 12 o'clock | Relative to max. direction | 3 o’clock | Relative to max. direction | 6 o’clock | Relative to max. direction | 9 o’clock | Relative to max. direction | p-value* | Illuminance  Skew direction** | 12 o'clock | Relative to max. direction | 3 o’clock | Relative to max. direction | 6 o’clock | Relative to max. direction | 9 o’clock | Relative to max. direction | p-value* | Illuminance Skew direction** |
| Storz Flex-Xc | 4693 (4592 to  4794) | 81% | 5827 (4913 to 6740) | 100% | 5434 (5059 to 5809) | 93% | 4377 (4250 to 4504) | 75% | p <0.001 | 3 to 6 o’clock | 5155 (4937 to 5372) | 87% | 5951 (4947 to 6955) | 100% | 5223 (4977 to 5469) | 88% | 4242 (4165 to 4318) | 71% | p <0.001 | 12 to 6 o’clock |
| Storz Flex-X2s | 1593 (1474 to 1712) | 100% | 1373 (1291 to 1456) | 86% | 1277 (1114 to 1439) | 80% | 1407 (1363 to 1451) | 88% | p <0.001 | 9 to 12 o’clock | 2155 (2043 to 2267) | 100% | 1923 (1833 to 2013) | 89% | 1819 (1600 to 2038) | 84% | 2051 (2008 to 2093) | 95% | p <0.001 | 9 to 12 o’clock |
| Olympus V3 | 226 (167 to 286) | 100% | 219 (159 to 278) | 97% | 197 (189 to 204) | 87% | 219 (200 to 239) | 97% | p =0.72 | None | 896 (713 to 1077) | 88% | 1015 (729 to 1301) | 100% | 864 (837 to 891) | 85% | 958 (891 to 1024) | 94% | p =0.52 | None |
| Olympus P7 | 796 (680 to 912) | 100% | 791 (667 to 916) | 99% | 531 (474 to 588) | 67% | 514 (456 to 571) | 65% | p <0.001 | 12 to 3 o’clock | 1911 (1631 to 2191) | 100% | 1833 (1583 to 2083) | 96% | 1261 (1188 to 1333) | 66% | 1191 (1088 to 1295) | 62% | p <0.001 | 12 to 3 o’clock |
| Pusen 7.5F | 179 (163 to 194) | 100% | 155 (144 to 167) | 87% | 134 (120 to 149) | 75% | 149 (131 to 166) | 83% | p <0.001 | 12 to 3 o’clock | 300 (267 to 333) | 100% | 243 (225 to 261) | 81% | 216 (193 to 239) | 72% | 239 (212 to 265) | 80% | p <0.001 | 12 o’clock |
| Pusen 9.2F | 917 (697 to 1138) | 100% | 837 (676 to 998) | 91% | 619 (587 to 651) | 67% | 662 (632 to 692) | 72% | p <0.01 | 12 to 3 o’clock | 1420 (1130 to 1709) | 100% | 1340 (1114 to 1567) | 94% | 1091 (1029 to 1152) | 77% | 1077 (1010 to 1143) | 76% | p =0.01 | 12 to 3 o’clock |
| OTU WiScope | 233 (220 to 247) | 86% | 254 (243 to 266) | 93% | 272 (266 to 279) | 100% | 263 (244 to 282) | 97% | p <0.001 | 3 to 9 o’clock | 447 (430 to 464) | 85% | 491 (477 to 505) | 93% | 527 (521 to 533) | 100% | 510 (476 to 545) | 97% | p <0.001 | 6 to 9 o’clock |
| *ANOVA comparing all peripheral illuminance directions  **Direction of maximum illuminance and other directions not significantly different to the maximum illuminance (Tukey’s multiple comparisons)  Max. = Maximum | | | | | | | | | | | | | | | | | | | | |
