# Supplementary material for: Illumination matters Part II: advanced comparative analysis of flexible ureteroscopes in a kidney model by PEARLS
Source: World J Urol. 2024 May 6;42(1):298. doi: 10.1007/s00345-024-04987-2 (PMC11074033; doi:10.1007/s00345-024-04987-2)
Supplement: Supplementary file 3 — Supplementary file3 (DOCX 18 KB) [file 345_2024_4987_MOESM3_ESM.docx]

| **Supplementary Table 2. Color temperature measurements of ureteroscopes in a pink kidney calyx model** | | | | | | | | | | | | | | | | | | | | | |
| --- | --- | --- | --- | --- | --- | --- | --- | --- | --- | --- | --- | --- | --- | --- | --- | --- | --- | --- | --- | --- | --- |
| Scope | Mean colour temperature (Kelvin) | | | | | | | | | | | | | | | | | | | | |
|  | 50% brightness setting | | | | | | | | | | 100% brightness setting | | | | | | | | | | |
|  | Overall (95% CI) | Centre  (95% CI) | 45 ° opening positions  (95% CI) | 90° opening positions  (95% CI) | Mean change center vs 45° (95% CI) | % change | p-value* | Mean change center vs 90°  (95% CI) | % change | p-value** | Overall (95% CI) | Centre  (95% CI) | 45 ° opening positions  (95% CI) | 90° opening positions  (95% CI) | Mean change center vs 45° (95% CI) | % change | p-value* | Mean change center vs 90°  (95% CI) | % change | p-value** |  |
| Storz Flex-Xc | 3956 (3760 to 4152) | 5347 (5305 to 5388) | 3986 (3720 to 4253) | 3579 (3552 to 3605) | -1361(-1897 to -824.6) | -25% | p <0.01 | -1768 (-1824 to -1713) | -33% | p <0.01 | 3987 (3794 to 4180) | 5300 (5240 to 5360) | 4066 (3803 to 4329) | 3579 (3550 to 3609) | -1234 (-1763 to -705.5) | -23% | p <0.01 | -1721 (-1784 to -1658) | -32% | p <0.01 |  |
| Storz Flex-X2s | 3909 (3750 to 4069) | 5380 (5236 to 5525) | 3718 (3671 to 3765) | 3733 (3720 to 3746) | -1663 (-1770 to -1555) | -31% | p <0.01 | -1647 (-1704 to -1590) | -31% | p <0.01 | 3924 (3764 to 4085) | 5409 (5297 to 5521) | 3738 (3708 to 3768) | 3740 (3724 to 3756) | -1671 (-1743 to -1598) | -31% | p <0.01 | -1669 (-1719 to -1619) | -31% | p <0.01 |  |
| Olympus V3 | 2922 (2745 to 3099) | 4409 (4333 to 4486) | 2901 (2766 to 3036) | 2571 (2530 to 2613) | -1508 (-1781 to -1236) | -34% | p <0.01 | -1838 (-1925 to -1751) | -42% | p <0.01 | 3075 (2877 to 3274) | 4778 (4677 to 4879) | 3031 (2897 to 3164) | 2694 (2648 to 2741) | -1747 (-2018 to -1477) | -37% | p <0.01 | -2084 (-2183 to -1984) | -44% | p <0.01 |  |
| Olympus P7 | 3160 (2853 to 3468) | 5907 (5727 to 6087) | 2982 (2816 to 3148) | 2652 (2612 to 2692) | -2925 (-3264 to -2586) | -50% | p <0.01 | -3255 (-3356 to -3153) | -55% | p <0.01 | 3121 (2915 to 3327) | 4889 (4798 to 4980) | 3043 (2884 to 3201) | 2758 (2724 to 2792) | -1846 (-2166 to -1526) | -38% | p <0.01 | -2131 (-2206 to -2056) | -44% | p <0.01 |  |
| Pusen 7.5F | 4592 (4389 to 4794) | 6051 (5886 to 6215) | 4560 (4275 to 4844) | 4259 (4245 to 4273) | -1491 (-2065 to -916.3) | -25% | p <0.01 | -1792 (-1855 to -1728) | -30% | p <0.01 | 4502 (4223 to 4781) | 6138 (5972 to 6303) | 4338 (3825 to 4850) | 4257 (4244 to 4270) | -1800 (-2831 to -768.8) | -29% | p <0.01 | -1881 (-1944 to -1817) | -31% | p <0.01 |  |
| Pusen 9.2F | 4110 (3814 to 4406) | 6787 (6574 to 7000) | 3927 (3807 to 4047) | 3624 (3598 to 3650) | -2860 (-3112 to -2608) | -42% | p <0.01 | -3163 (-3253 to -3072) | -47% | p <0.01 | 4045 (3693 to 4397) | 6945 (6486 to 7403) | 3900 (3824 to 3976) | 3466 (3122 to 3809) | -3045 (-3265 to -2825) | -44% | p <0.01 | -3479 (-4188 to -2770) | -50% | p <0.01 |  |
| OTU WiScope | 3969 (3834 to 4104) | 5125 (4996 to 5255) | 3896 (3785 to 4006) | 3753 (3739 to 3767) | -1230 (-1456 to -1003) | -24% | p <0.01 | -1373 (-1426 to -1320) | -27% | p <0.01 | 3960 (3800 to 4119) | 5410 (5326 to 5494) | 3818 (3742 to 3893) | 3739 (3730 to 3748) | -1592 (-1747 to -1438) | -29% | p <0.01 | -1671 (-1705 to -1637) | -31% | p <0.01 |  |
| * Student’s t-test comparing center vs 45°  ** Student’s t-test comparing center vs 90° | | | | | | | | | | | | | | | | | | | | | |
